# Secular Trends in Lipid Profiles in Korean Adults Based on the 2005–2015 KNHANES

**DOI:** 10.3390/ijerph16142555

**Published:** 2019-07-17

**Authors:** Yu-Jin Kwon, Jae-Woo Lee, Hee-Taik Kang

**Affiliations:** 1 Department of Family Medicine, Yonsei University College of Medicine, Seoul 03722, Korea; 2 Department of Medicine, Graduate School of Yonsei University, Seoul 03722, Korea; 3 Department of Family Medicine, Yong-In Severance Hospital, Yong-In 17046, Gyounggi, Korea; 4 Department of Family Medicine, Chungbuk National University Hospital, Cheongju 28644, Chungbuk, Korea; 5 Department of Family Medicine, Chungbuk National University College of Medicine, Cheongju 28644, Chungbuk, Korea

**Keywords:** total cholesterol, triglyceride, high-density lipoprotein cholesterol, dyslipidemia, prevalence

## Abstract

Dyslipidemia is a primary, critical risk factor for cardiovascular disease. Therefore, evaluating the trends in lipid profiles is crucial for the development of health policies and programs. We studied trends in lipid profiles in Korean adults over an 11-year period according to the use of lipid-lowering medications through age-specific analysis. A total of 73,890 participants were included in the Korean National Health and Nutrition Examination Survey III (2005)-VI (2013–2015). The proportion of participants on lipid-lowering medications has increased. This trend was apparent in age groups of over 40 years in both men and women. Lipid-lowering medications successfully reduced mean total cholesterol (TC), but there was no favorable trend in TC in participants not taking lipid-lowering medication in both men and women. Unlike men, triglyceride and non-high-density lipoprotein cholesterol (HDL) decreased in women without lipid-lowering medications. In age-specific hypercholesterolemia, the prevalence of hypercholesterolemia significantly increased in the age groups of 30–59 and 30–49 years in men and women without lipid-lowering medications, respectively. Meanwhile, mean HDL-C levels increased over the 11-year period regardless of lipid-lowering drug use in both men and women. These analyses identified an upward trend in TC and HDL-C over the 11-year period.

## 1. Introduction

According to the 2017 statistical update of the American Heart Association, the number of deaths from cardiovascular disease (CVD) has been decreasing since 2004 [1]. However, CVD remains the leading cause of total mortality worldwide, accounting for 30% of all global deaths [1,2]. CVD is responsible for an immense socioeconomic burden on patients and society in general. There are several methods to prevent CVD, including smoking cessation, increased physical activity, and adoption of a healthy diet [3,4]. Dyslipidemia, i.e., abnormal lipid levels in the blood, is considered one of the most important modifiable risk factors of CVD [5]. One recent study found that even moderate hyperlipidemia increases one’s risk of coronary heart disease (CHD) later in life, depending on the length of exposure [6]. Therefore, active screening and treatment of dyslipidemia are the first steps in prevention of CVD. Many countries have established public health programs to address dyslipidemia [7]. These efforts have successfully led to a decrease in the prevalence of hypercholesterolemia in 1999–2000 and 2013–2014 in the United States [8]. In Europe, mean total cholesterol (TC) levels have also declined over the last 30 years [9]. However, these analyses were only based on serum cholesterol concentrations, without considering whether participants were prescribed lipid-lowering drugs. Meanwhile, the prevalence of hypercholesterolemia continues to increase in Japanese and Turkish populations [10,11], concurrently with a trend of rising TC and low-density lipoprotein cholesterol (LDL-C) levels in China [12]. One Korean study reported that the prevalence of dyslipidemia is declining [13]; however, there is a need for more recent data with regard to lipid profiles to establish appropriate public health policies. There is also a need for studies that address the use of lipid-lowering drugs.

Therefore, we aimed to investigate the 11-year trends regarding changes in the lipid profile of participants after stratifying for lipid-lowering medication use by age-specific analysis.

## 2. Materials and Methods

### 2.1. Study Subjects

The Korean National Health and Nutrition Examination Survey (KNHANES), a cross-sectional national representative survey, was conducted by the Korea Centers for Disease Control and Prevention (KCDC) based on the National Health Promotion Act. This survey uses complex and multistep probability sample design to obtain a representative sample of the civilian, non-institutionalized Korean population. The primary sample unit (PSU) is selected from geographical data from the entire country, and 20 final target households were sampled for each PSU using systematic sampling. Approximately 10,000 samples were collected for approximately 192 PSUs per year. Statistical weights were assigned to each participant to represent the entire Korean population. This survey is composed of three component surveys (a health interview, health examination, and nutrition survey) in the six phases of KNHANES I (1998), II (2001), III (2005), IV (2007–2009), V (2010–2012), and VI (2013–2015). These data provide a variety of information regarding health status, health behavior, socioeconomic demographics, and laboratory tests. Trained interviewers conducted face-to-face interviews with participants. Participants could refuse to participate in this survey, and a written agreement was obtained by participants upon consent [14]. Detailed information about KNHANES has been published in a previous study by the KCDC [14,15].

Due to laboratory data inaccuracies in the KNHANES I and II surveys, we used data from the KNHANES III (2005), IV (2007–2009), V (2010–2012), and VI (2013–2015). Of the total 107,498 participants from the KNHANES III–VI surveys, we excluded individuals meeting any of the following criteria: <20 years of age, patients who had not fasted for at least 8 hours prior to sampling, and patients who had missing values on a health interview survey regarding dyslipidemia. After these exclusions, 73,935 participants (32,260 men and 41,675 women) were ultimately included in this study. The Institutional Review Board (IRB) of the KCDC approved this study.

The ethical approval code number of Institutional Review Board (IRB) of the Korea Centers for Disease Control and Prevention (KCDC) is 2013-12EXP-03-5C.

### 2.2. Anthropometric and Laboratory Measurements

Well-trained staff obtained anthropometric measurements following standard procedures. Body weight was measured to the nearest 0.1 kg, and height and waist circumference (WC) were measured to the nearest 0.1 cm while the participants were wearing light clothing and no shoes. The body mass index (BMI) was calculated as the ratio of weight (kg) to height (m^2^) (kg/m^2^). Systolic and diastolic blood pressure (SBP and DBP, respectively) were obtained using a standard mercury sphygmomanometer with participants in a seated position. Blood samples were collected from an antecubital vein in the morning after an overnight fast. The serum levels of TC, high-density lipoprotein cholesterol (HDL-C), triglyceride (TG), and fasting plasma glucose (FPG) were measured enzymatically using an Advia 1650/2400 (Siemens, New York, NY, USA) in the 2005 and 2007 surveys, and a Hitachi Automatic Analyzer 7600/7600-210 (Hitachi, Tokyo, Japan) in the 2008, 2010, 2011, 2012, 2013, 2014, and 2015 surveys.

### 2.3. Lipid Profiles, and Related Variables

According to the criteria of the National Cholesterol Education Program Adult Treatment Panel III (NCEP-ATP III) [16], hypercholesterolemia is defined as serum TC ≥ 240 mg/dL; hypertriglyceridemia is defined as TG ≥ 150 mg/dL; and hypo-HDL-cholesterolemia is defined as serum HDL-C < 40 mg/dL in men and < 50 mg/dL in women. Non-HDL cholesterol was calculated by subtracting the HDL-C value from a TC value [17]. Education duration was divided into four groups: <6 years, 6–9 years, 9–12 years, and ≥12 years. Occupational status was categorized as manual work (clerk, service, and sales workers, skilled agricultural, forestry and fishery workers, and persons who operate or assemble equipment or machines), office work (general managers, government administrators, professionals, and simple office worker), or other (unemployed persons, housekeepers, and students).

### 2.4. Statistical Analysis

All data for continuous variables are presented as means ± standard error (SE). Data for categorical variables are presented as percentages ± SE. All sampling and weight variables were stratified. The SAS survey was used for statistical analysis to account for the complex sampling design and to provide nationally representative prevalence estimates. Analysis of variance (ANOVA) was used to compare the mean values of the continuous variables, analysis of covariance (ANCOVA) was used to compare the age-adjusted lipid level across the KNHANES phases, and the *x^2^* test was used to compare categorical variables. *P* for trend values were calculated among the KNHANES phases by logistic regression analyses or linear regression after setting the phase as the continuous variable. We conducted Bonferroni correction for multiple testing. Statistical analyses were performed using SAS version 9.4 (SAS Institute Inc., Cary, NC, USA).

## 3. Results

### 3.1. Characteristics of Sample

The unweighted sample sizes for this study in the KNHANES III (2005), IV (2007–2009), V (2010–2012), and VI (2013–2015) were 24,752, 16,359, 17,243, and 15,536, respectively, and the mean ages were 43.6, 44.8, 45.7, and 46.6 years, respectively (Table 1). The number and proportion of women and men participating in the study were 13,251 (53.5%) and 11,501 (46.5%) in phase III, 9454 (57.8%) and 6905 (42.2%) in phase IV, 9994 (57.9%) and 7249 (42.1%) in phase V, and 8976 (57.8%) and 6560 (42.2%) in phase VI, respectively.

Table 2 describes the characteristics of participants after stratifying by sex and use of lipid-lowering medications. Age significantly increased across the KNHANES phases. There were decreasing trends in BMI in men and women with lipid-lowering medication use (β-coefficient = −0.354 and *p* for trend < 0.001 in men, β-coefficient = −0.363 and *p* for trend < 0.001 in women). The level of BMI increased in men without lipid-lowering medication use (β-coefficient = 0.107 and p for trend < 0.001), while BMI showed no difference in women without lipid-lowering medication use over time.

### 3.2. Lipid Profiles

Table 3 shows the changes in mean lipid profile levels according to sex and age groups across the KNHANES phases. The mean TC levels increased linearly from 183.4 mg/dL to 187.7 mg/dL (β-coefficient = 1.331, *p* for trend < 0.001) in men, and from 184.3 mg/dL to 187.9 mg/dL (β-coefficient = 0.805, *p* for trend = 0.002) in women. The mean HDL-C levels increased over the 11-year period in both men and women (β-coefficient = 1.446, *p* for trend < 0.001 and β-coefficient = 2.308, *p* for trend < 0.001), respectively.

### 3.3. Lipid-lowering Medications

**Figure 1:**
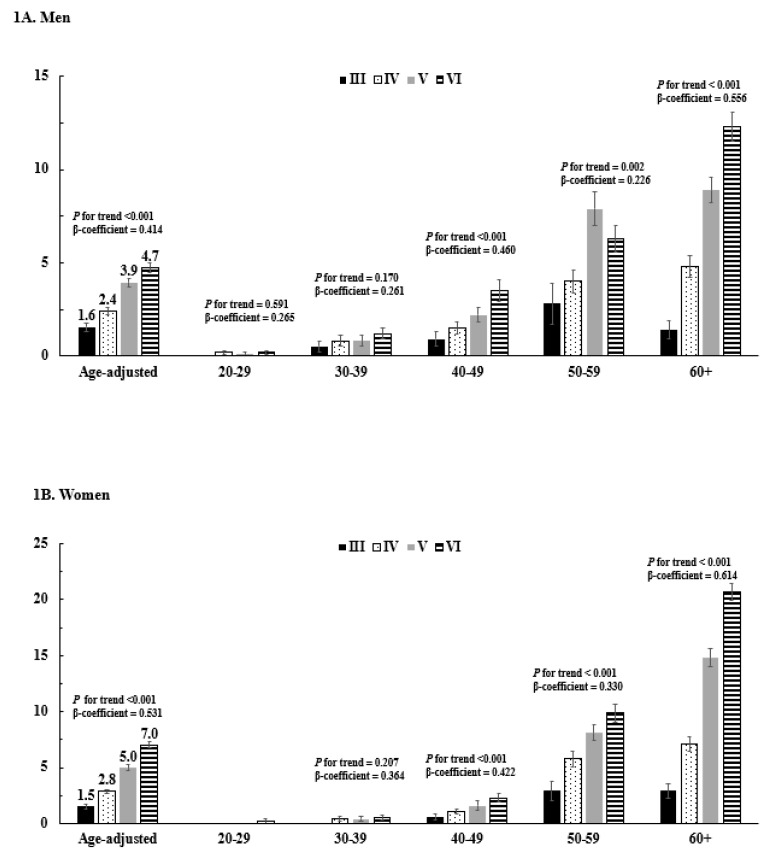
Age-adjusted and age-specific prevalence of use of lipid-lowering medication. (**A**) Men; (**B**) women. *p*-values for trends were determined by logistic regression analyses after setting the KNHANES phase as the continuous variable. KNHANES: Korean National Health and Nutrition Examination Survey.

Figure 1 presents the age-adjusted and age-specific proportion of those taking lipid-lowering medications across the KNHANES phases. For KNHANES III–VI (2005 and 2007–2015), the proportion of participants on lipid-lowering medication increased from 1.6 to 4.7% in men (β-coefficient = 0.414, *p* for trend < 0.001) (Figure 1A) and from 1.5 to 7% in women (β-coefficient = 0.531, *p* for trend < 0.001) (Figure 1B). This trend was apparent in age groups over 40 years in both men and women.

### 3.4. Lipid Profile According to Treatment of Lipid Lowering Drug

To clarify the effect of lipid-lowering medications, we also investigated lipid profile trends in participants after stratifying by use of lipid-lowering drugs (Table 4). The mean TC and non-HDL- C levels declined in men with lipid-lowering drug use (TC: 195.7 to 174.6 mg/dL, *p* for trend < 0.001; non-HDL-C: 240.4 to 213.0 mg/dL, *p* for trend < 0.001) over time. Mean TC and TG levels increased in men without lipid-lowering drug use (TC: 183.6 to 188.9 mg/dL, *p* for trend < 0.001; TG: 155.0 to 162.8 mg/dL, *p* for trend = 0.002). In women with lipid-lowering drug use, all lipid profiles levels, except HDL-C, decreased over time. TC was significantly elevated in women without lipid-lowering drug use (TC: 184.4 to 190.1 mg/dL, *p* for trend < 0.001), while other lipid profiles (TG and non-HDL-C) decreased during the same time period.

**Figure 2:**
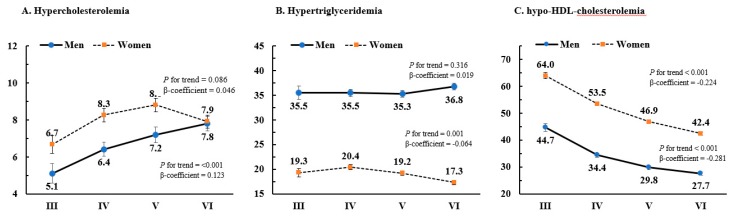
Trends in age-adjusted prevalence of hypercholesterolemia, hypertriglyceridemia, and hypo-HDL-cholesterolemia in subjects with no use of lipid-lowering medication. (**A**) Hypercholesterolemia. (**B**) Hypertriglyceridemia. (**C**) Hypo-HDL-cholesterolemia. *P*-values for trends were determined by logistic regression analyses after setting the KNHANES phase as the continuous variable. KNHANES: Korean National Health and Nutrition Examination Survey.

Figure 2 presents the age-adjusted prevalence of hypercholesterolemia (A), hypertriglyceridemia (B), and hypo-HDL-cholesterolemia (C) in participants without lipid-lowering medication use. There was an increasing trend in hypercholesterolemia in men (β-coefficient = 0.123 and *p* for trend < 0.001) over the examination cycles. By contrast, the prevalence of hypercholesterolemia was not significantly different, and the prevalence of hypertriglyceridemia slightly decreased over time (β-coefficient = −0.064 and *p* for trend < 0.001) in women. There were significant downward trends in the prevalence of hypo-HDL-cholesterolemia in both men and women (β-coefficient = −0.281 for men, −0.224 for women, and *p* for trend < 0.001 in both sexes) over the time course of the study period.

**Figure 3:**
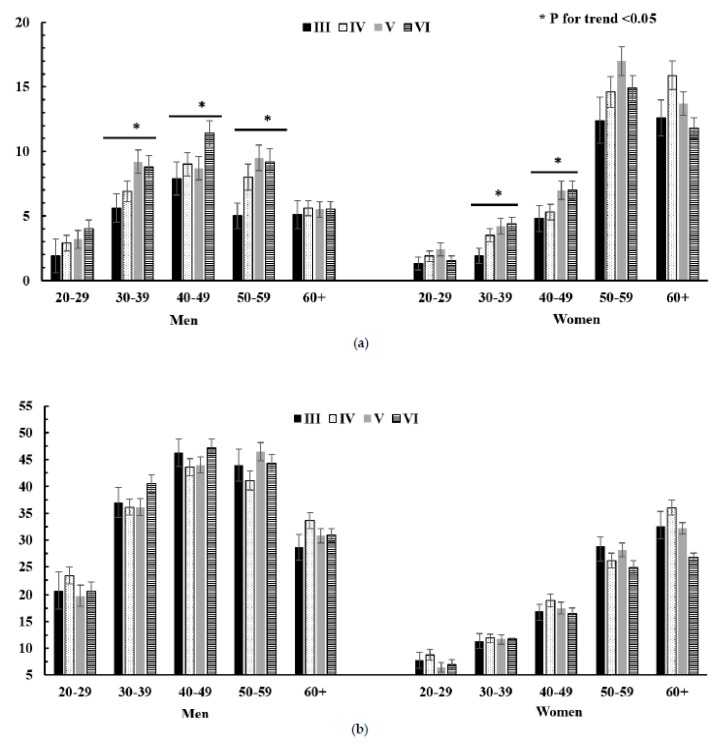

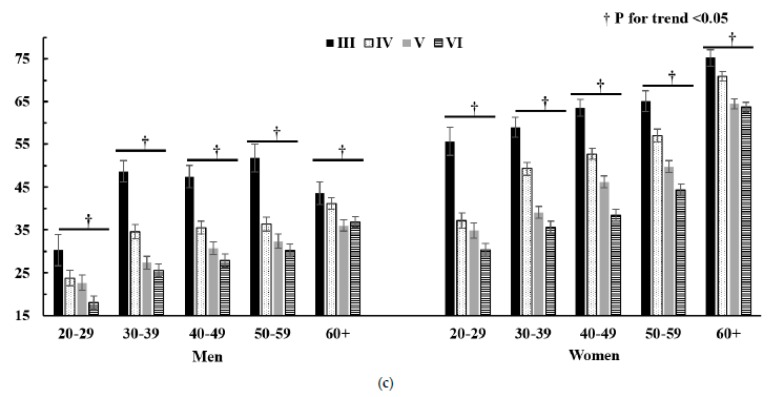
Trends in age-specific prevalence of hypercholesterolemia, hypertriglyceridemia, and hypo-HDL-cholesterolemia in subjects with no use of lipid-lowering medication. (**a**) Hypercholesterolemia; (**b**) hypertriglyceridemia; (**c**) hypo-HDL-cholesterolemia.

In age-specific hypercholesterolemia, the prevalence of hypercholesterolemia significantly increased in the age groups of 30–59 and 30–49 years in men and women, respectively, without lipid-lowering medication use (Figure 3a). The prevalence of hypertriglyceridemia was not significantly different in all age groups in men and women (Figure 3b). The prevalence of hypo-HDL cholesterolemia significantly decreased in all age groups of men and women (Figure 3c).

## 4. Discussion

Our data revealed that TC and HDL-C level increased and the proportion of taking lipid-lowering medications increased over the 11-year study period.

Many clinical and epidemiologic studies have demonstrated that dyslipidemia is an important risk factor of CVD [6,18,19]. Therefore, many health authorities, including those in Korea, have conducted national health screening programs for early detection and management of dyslipidemia [20,21]. The National Cholesterol Expert Panel (NCEP) suggests screening for dyslipidemia in adults over 20 years of age [22], while the United States Preventive Services Task Force (USPSTF) provides positive evidence for screening dyslipidemia in adults [23]. Based on standard guidelines, expert groups also recommend active management, such as therapeutic lifestyle modification and use of appropriate medications (such as statins) in patients with dyslipidemia [21,22,24].

In this study, we determined that the proportion of adults on lipid-lowering drugs gradually increased over the course of KNHANES phases III–VI, and that the proportion of participants on lipid-lowering medication was higher in women than men. Depending on the use of lipid-lowering drug, the lipid profile in participants taking lipid-lowering medications improved, with decreasing TC, non-HDL-C, and TG and increasing HDL-C levels in both men and women. Meanwhile, TC increased in participants who were not taking lipid-lowering drugs. HDL-C level consistently increased regardless of sex, age, and use of lipid-lowering drug. Age-specific analysis revealed an upward trend in TC that was significant in the 30–59 years age group in men and the 30–49 years age group in women without lipid-lowering medication use. The level of lipid-lowering drug usage did not increase significantly in the relatively younger age groups.

Our findings are in agreement with several previous studies. Several studies identified the increasing trend of TC in Japan, Turkey, and China [10,11,12]. Meanwhile, a recent Japanese study reported that serum HDL-C continues to increase using the National Health and Nutrition Survey [25]. A study conducted in a Canadian province also revealed a significant downward trend in the prevalence of hypo-HDL-cholesterolemia over a six-year period [26]. There is no exact reason to explain such trends. HDL-C levels are affected not only by genetic factors, but also by socioeconomic status (i.e., education level, economic level, and occupation) [27].

High socioeconomic status has been regarded as a key determinant of medical intervention accessibility and exposure to modifiable risk factors [28]. The Global Burden of Disease (GBD) 2015 study reported that the prevalence of CVD has declined in regions with a high socioeconomic index (including per capita income and education level) [28]. Korea is one of the most developed countries in Asia, and both household incomes and education levels have improved between 2005 and 2015. Favorable changes in socioeconomic status may lead to increased HDL-C levels.

TG in women without anti-dyslipidemic use has declined. The differences in TG trends between men and women may partly be explained by different rates of obesity between the sexes. According to our previous study on obesity trends, male obesity has increased with time, while female obesity has leveled off, or even decreased [29]. Different trends in obesity prevalence between men and women may also subsequently lead to variable lipid profiles in participants who are not taking lipid-lowering drugs.

TC level has been increasing in adults (30–59 years) not taking lipid-lowering medication. These results suggest that more active and practical interventions are needed to prevent and manage dyslipidemia in these age groups. Screening obese individuals and those with unhealthy lifestyles should be the first step to prevent dyslipidemia and reduce future CVD events [30,31,32]. Many individuals are unaware that they have dyslipidemia. Although the awareness rate of dyslipidemia varies according to prior studies, one recent Korean study reported that only approximately 17% of adults with dyslipidemia over 20 years old were aware of their dyslipidemia status [33]. Effective medical therapy, such as administration of statin drugs, helps to prevent CVD [34,35]. However, recent studies suggested that chronic inflammation is a key risk factor of CVD [36]. Therefore, reducing inflammation manifestation by lifestyle modifications, such as increasing physical activity, adopting healthy diet, and maintaining a healthy body weight, must also be emphasized [4,36].

This study had several limitations. First, we did not include the low-density lipoprotein cholesterol (LDL-C) levels because they were not directly measured in KNHANES. Although LDL-C levels estimated by the Friedwald equation were correlated with direct measurement, underestimation of the LDL-C level is an important problem to evaluate the high-risk group in the presence of TG ≥ 150 mg/dL [37]. Therefore, we did not include LDL levels to minimize bias. Alternatively, we considered non-HDL cholesterol. Several studies supported that non-HDL-C could be a more reliable predictor for CVD [38,39]. Second, we did not distinguish between the types of lipid-lowering medications used by patients. Different drug classes (such as statins, fibric acid, and niacin) have variable effects on specific lipid levels, and in particular on HDL-C [40]. Therefore, the HDL-C level may have been influenced by the type of drug used. Furthermore, we could not consider HDL functionality. Emerging evidence established that HDL functionality is more important than quantity, and HDL-C may no longer be a reliable marker [41,42]. Our authors believe that there is a need to develop inexpensive methods to evaluate functionality of HDL using a large population-based study. Finally, there could have been bias deriving from unequal sex ratio.

Despite these limitations, our study has several advantages. First, we applied sampling weights to all analyses to represent the average Korean citizen. Second, this study investigated an 11-year trend of lipid profiles using nationally representative data. Finally, we examined the lipid profile trend in patients who were and were not treated with lipid-lowering medications.

## 5. Conclusions

The analyses identified an upward trend in TC and HDL-C over the 11-year period. There was also an increase in the use of lipid-lowering drugs over time. The positive trends in TC level were only seen in adults who used lipid-lowering drugs. We must continually monitor how long these trends continue, and what type of changes occur. Furthermore, more active strategies for lifestyle changes, as well as more intense screening and treatment of dyslipidemia, remain very important in the reduction of CVD risk in Korean adults.

## Figures and Tables

**Table 1:** Study population according to Korean National Health and Nutrition Examination Survey (KNHANES) phase, age group, and sex.

Age Group	III (2005)	IV (2007–2009)	V (2010–2012)	VI (2013–2015)
Total, unweighted *N*	24752	16359	17243	15536
Age (years, mean ± SE)	43.6 ± 0.4	44.8 ± 0.3	45.7 ± 0.2	46.6 ± 0.2
Men, unweighted *N* (%)	11501 (46.5)	6905 (42.2)	7249 (42.1)	6560 (42.2)
Age (years, mean ± SE)	42.8 ± 0.4	43.8 ± 0.4	44.7 ± 0.3	45.5 ± 0.3
20–29 years (%)	21.7 (1.5)	20.2 (0.9)	19.0 (0.8)	18.7 (0.7)
30–39 years (%)	25.0 (1.4)	23.3 (0.9)	21.6 (0.7)	20.0 (0.7)
40–49 years (%)	23.8 (1.1)	23.4 (0.8)	23.0 (0.7)	21.6 (0.6)
50–59 years (%)	14.7 (0.9)	16.7 (0.6)	18.8 (0.6)	20.2 (0.6)
≥60 years (%)	14.9 (0.8)	16.3 (0.6)	17.5 (0.6)	19.6 (0.6)
Women, unweighted *N* (%)	13251 (53.5)	9454 (57.8)	9994 (57.9)	8976 (57.8)
Age (years, mean ± SE)	42.8 ± 0.4	43.8 ± 0.3	44.7 ± 0.3	45.5 ± 0.3
20–29 years (%)	20.5 (1.4)	18.4 (0.7)	17.0 (0.6)	15.9 (0.6)
30–39 years (%)	23.1 (1.1)	21.7 (0.7)	20.4 (0.6)	18.8 (0.6)
40–49 years (%)	22.3 (1.0)	22.1 (0.7)	21.6 (0.6)	21.1 (0.6)
50–59 years (%)	14.3 (0.7)	16.3 (0.5)	18.6 (0.5)	20.0 (0.5)
≥60 years (%)	19.9 (0.9)	21.4 (0.7)	22.6 (0.6)	24.2 (0.6)

All data are presented as mean ± standard error (SE) or percentage (SE).

**Table 2:** Participant characteristics according to KNHANES phase according to use of lipid-lowering medications.

**With Lipid-lowering Medication**								
	**Men**				**Women**			
**KNHANES Phase**	**III**	**IV**	**V**	**VI**	**III**	**IV**	**V**	**VI**
N (%)	110 (1.0)	175 (2.5)	380 (5.2)	437 (6.7)	177 (1.3)	349 (3.7)	686 (6.9)	889 (9.9)
Age (years) *^,^†	52.4 ± 1.9	55.5 ± 1.0	57.7 ± 0.7	58.8 ± 0.7	59.9 ± 1.2	60.7 ± 0.7	62.6 ± 0.5	63.5 ± 0.4
BMI (kg/m²) *^,^†	25.9 ± 0.4	25.8 ± 0.3	25.4 ± 0.2	25.0 ± 0.2	25.1 ± 0.3	25.8 ± 0.3	25.6 ± 0.2	24.9 ± 0.1
WC (cm) *^,^†	91.4 ± 2.7	90.1 ± 0.7	89.3 ± 0.6	88.1 ± 0.5	85.6 ± 0.8	86.7 ± 0.7	85.8 ± 0.6	83.8 ± 0.4
SBP (mmHg) *^,^†	131.8 ± 2.5	128.4 ± 1.6	124.9 ± 0.9	123.0 ± 0.7	124.9 ± 3.0	127.7 ± 1.0	128.5 ± 0.8	125.7 ± 0.7
DBP (mmHg) *^,^†	84.5 ± 1.5	83.3 ± 0.9	79.5 ± 0.6	77.0 ± 0.5	76.5 ± 1.7	79.6 ± 0.6	75.8 ± 0.5	74.5 ± 0.4
FPG (mg/dL) †	107.2 ± 3.4	112.2 ± 2.7	115.0 ± 2.1	111.9 ± 1.4	111.3 ± 5.2	109.6 ± 2.0	110.0 ± 1.8	107.9 ± 1.1
Monthly household income *^,^†	2650 ± 318	3647 ± 825	4737 ± 709	3851 ± 181	1814 ± 178	3732 ± 104.4	3520 ± 425	3005 ± 141
Occupation (%)								
Office worker *^,^†	35.7 ± 10.3	32.0 ± 4.3	20.5 ± 2.7	24.7 ± 2.5	2.4 ± 0.2	4.5 ± 2.0	3.7 ± 1.0	6.6 ± 1.0
Manual worker *^,^†	45.0 ± 7.6	32.3 ± 4.1	46.2 ± 3.4	42.3 ± 2.8	27.2 ± 4.4	25.1 ± 3.0	26.5 ± 2.2	29.7 ± 1.8
Other†	19.3 ± 7.4	35.7 ± 4.1	33.3 ± 3.1	33.0 ± 2.7	70.4 ± 4.4	70.4 ± 3.2	70.0 ± 2.2	63.7 ± 1.9
Education duration (%)								
<6 years†	21.4 ± 6.3	13.9 ± 2.7	22.0 ± 2.8	18.7 ± 2.1	63.0 ± 6.7	57.0 ± 3.5	60.1 ± 2.5	51.8 ± 2.1
6–8 years *^,^†	11.2 ± 4.6	20.6 ± 4.0	19.6 ± 2.6	14.9 ± 2.0	10.4 ± 4.7	15.8 ± 2.7	14.2 ± 1.7	15.0 ± 1.5
9–11 years †	48.4 ± 8.9	27.5 ± 3.9	32.9 ± 3.3	34.5 ± 2.9	17.0 ± 3.2	18.4 ± 2.5	20.1 ± 2.1	22.4 ± 1.6
≥12 years †	18.9 ± 7.8	37.9 ± 5.2	25.4 ± 2.8	31.9 ± 2.8	9.6 ± 4.2	8.8 ± 2.5	5.7 ± 1.1	10.9 ± 1.3
**Without lipid-lowering medication**								
	**Men**				**Women**			
**KNHANES Phase**	**III**	**IV**	**V**	**VI**	**III**	**IV**	**V**	**VI**
N (%)	11391 (99.0)	6730 (97.5)	6869 (94.8)	6123 (93.3)	13074 (96.3)	9105 (93.1)	9308 (93.1)	8087 (90.1)
Age (years) *^,^†	42.7 ± 0.4	43.5 ± 0.3	44.2 ± 0.3	44.9 ± 0.3	44.3 ± 0.4	45.4 ± 0.3	45.9 ± 0.3	46.3 ± 0.3
BMI (kg/m²) *	23.9 ± 0.1	24.0 ± 0.1	24.1 ± 0.1	24.3 ± 0.0	23.3 ± 0.1	23.2 ± 0.0	23.2 ± 0.1	23.1 ± 0.0
WC (cm) *^,^†	83.6 ± 0.3	84.2 ± 0.2	84.0 ± 0.2	84.7 ± 0.1	77.9 ± 0.3	78.3 ± 0.2	77.6 ± 0.2	77.4 ± 0.2
SBP (mmHg)	121.0 ± 0.4	119.0 ± 0.3	120.6 ± 0.3	119.1 ± 0.2	114.7 ± 0.5	113.4 ± 0.3	115.4 ± 0.3	113.4 ± 0.3
DBP (mmHg) *^,^†	80.2 ± 0.4	79.5 ± 0.2	79.4 ± 0.2	77.8 ± 0.2	74.1 ± 0.3	73.5 ± 0.2	73.7 ± 0.1	72.4 ± 0.2
FPG (mg/dL) *^,^†	95.2 ± 0.5	97.1 ± 0.4	97.9 ± 0.3	100.4 ± 0.3	92.1 ± 0.5	94.1 ± 0.3	94.1 ± 0.3	95.9 ± 0.3
Monthly household income *^,^†	2442 ± 58	3108 ± 86	4703 ± 186	3961 ± 66	2374 ± 64	2914 ± 74	4404 ± 136	3769 ± 62
Occupation (%)								
Office worker *^,^†	24.5 ± 1.3	28.1 ± 0.8	29.5 ± 0.8	31.4 ± 0.9	15.1 ± 1.0	17.3 ± 0.6	19.9 ± 0.6	22.9 ± 0.6
Manual worker *	52.1 ± 1.5	48.5 ± 0.9	50.0 ± 0.9	45.3 ± 0.9	30.6 ± 1.2	28.7 ± 0.7	32.8 ± 0.7	29.2 ± 0.7
Other †	23.4 ± 1.2	23.4 ± 0.8	20.4 ± 0.7	23.4 ± 0.7	54.4 ± 1.3	53.9 ± 0.8	47.3 ± 0.7	47.9 ± 0.7
Education duration (%)								
<6 years *^,^†	11.8 ± 0.7	13.2 ± 0.6	11.6 ± 0.5	11.0 ± 0.5	26.0 ± 1.2	25.9 ± 0.8	23.6 ± 0.7	19.5 ± 0.7
6–8 years *^,^†	9.8 ± 0.7	10.3 ± 0.5	10.0 ± 0.5	8.4 ± 0.4	11.0 ± 0.7	10.4 ± 0.4	9.8 ± 0.4	9.1 ± 0.4
9–11 years *^,^†	43.3 ± 1.5	41.4 ± 1.0	41.2 ± 0.8	40.2 ± 0.9	38.1 ± 1.2	37.7 ± 0.8	35.8 ± 0.7	35.6 ± 0.7
≥12 years †	35.1 ± 1.5	35.1 ± 1.0	37.2 ± 0.9	40.4 ± 0.9	24.9 ± 1.4	26.0 ± 0.8	30.7 ± 0.8	35.8 ± 0.8

*p* for trend were determined by linear regression analysis with weighting of survey design. Age, BMI, WC, SBP, DBP, FPG, and monthly household income were presented as continuous values, and occupation and education level were presented as categorical values. Monthly household income: total monthly house income/rooted value of number of family members, 1 USD = 1000 Korean won. Occupation: office worker (general managers, government administrators, professionals, and office workers); manual worker (clerks, service and sales workers, skilled agricultural, forestry, and fishery workers, persons who operate or assemble craft, equipment, or machines, and elementary workers); other (unemployed persons, housekeepers, and students). Abbreviations: KNHANES: Korean National Health and Nutrition Examination Survey; BMI: body mass index; WC: waist circumference; SBP: systolic blood pressure; DBP: diastolic blood pressure; FPG: fasting plasma glucose. Ⅲ: 2005, Ⅳ: 2007–2009, Ⅴ: 2010–2012, Ⅵ: 2013–2015. Bonferroni correction for multiple testing was conducted; * *p* for trend < 0.0125 in men, † *p* for trend < 0.0125 in women.

**Table 3:** Trends of age-adjusted and age-specific mean lipid profiles according to sex.

KNHANES Phase	Men				Women			
	III	IV	V	VI	III	IV	V	VI
**TC (mg/dL)**								
Age-adjusted **‡	183.4 ± 0.9	185.5 ± 0.6	187.5 ± 0.6	187.7 ± 0.5	184.3 ± 0.7	187.7 ± 0.5	188.7 ± 0.5	187.9 ± 0.5
20–29 years ‡	173.3 ± 2.3	171.8 ± 1.4	174.6 ± 1.6	174.4 ± 1.2	165.4 ± 1.7	170.2 ± 1.2	170.5 ± 1.1	173.7 ± 1.0
30–39 years **‡	183.3 ± 1.7	188.0 ± 1.0	192.7 ± 1.1	193.6 ± 1.3	172.8 ± 1.4	175.7 ± 0.8	179.8 ± 1.0	180.1 ± 0.8
40–49 years ‡	191.7 ± 1.8	194.8 ± 1.2	194.1 ± 1.1	197.1 ± 1.1	183.3 ± 1.4	185.4 ± 0.8	189.0 ± 1.0	189.1 ± 0.9
50–59 years	187.3 ± 1.8	189.6 ± 1.3	193.2 ± 1.3	191.2 ± 1.1	200.4 ± 2.0	203.6 ± 1.3	205.1 ± 1.1	202.2 ± 0.9
60+ years ‡	183.1 ± 1.8	183.5 ± 1.1	182.9 ± 0.9	181.8 ± 0.9	200.2 ± 1.5	203.6 ± 1.1	198.9 ± 0.8	195.4 ± 0.9
**HDL-C (mg/dL)**								
Age-adjusted **‡	42.4 ± 0.3	45.5 ± 0.2	46.9 ± 0.2	47.6 ± 0.2	47.3 ± 0.3	50.7 ± 0.2	52.7 ± 0.2	54.8 ± 0.2
20–29 years ‡	44.5 ± 0.7	47.5 ± 0.4	48.7 ± 0.5	50.2 ± 0.4	49.7 ± 0.6	55.0 ± 0.4	56.2 ± 0.5	58.5 ± 0.4
30–39 years ‡	41.7 ± 0.4	44.7 ± 0.3	46.9 ± 0.4	47.4 ± 0.4	48.5 ± 0.5	51.7 ± 0.3	54.7 ± 0.4	56.6 ± 0.4
40–49 years ‡	42.0 ± 0.5	45.6 ± 0.4	46.0 ± 0.3	46.9 ± 0.4	47.3 ± 0.5	50.5 ± 0.3	52.4 ± 0.3	55.2 ± 0.3
50–59 years ‡	40.8 ± 0.6	45.1 ± 0.3	46.4 ± 0.4	46.7 ± 0.3	46.7 ± 0.6	49.9 ± 0.3	51.8 ± 0.3	53.8 ± 0.3
60+ years ‡	43.1 ± 0.6	44.6 ± 0.3	46.5 ± 0.3	46.7 ± 0.3	44.5 ± 0.4	46.5 ± 0.3	48.5 ± 0.3	50.0 ± 0.3
**TG (mg/dL)**								
Age-adjusted **‡	155.7 ± 3.6	152.9 ± 1.7	154.4 ± 2.0	163.5 ± 2.0	113.4 ± 1.5	113.6 ± 1.1	111.6 ± 1.2	109.3 ± 1.0
20–29 years	113.6 ± 5.5	120.3 ± 2.9	117.0 ± 4.3	120.5 ± 3.8	85.1 ± 2.6	84.1 ± 2.2	81.8 ± 1.8	81.4 ± 2.0
30–39 years	158.6 ± 8.2	156.3 ± 3.4	156.3 ± 4.0	176.2 ± 5.2	93.2 ± 2.3	94.1 ± 1.8	94.7 ± 2.2	94.7 ± 2.2
40–49 years	183.9 ± 8.6	177.4 ± 4.3	176.1 ± 4.9	150.2 ± 1.1	111.1 ± 3.4	109.7 ± 1.8	112.1 ± 4.1	106.2 ± 1.9
50–59 years	183.7 ± 9.2	166.8 ± 4.3	182.1 ± 5.2	179.1 ± 4.8	135.1 ± 5.5	131.7 ± 2.6	128.8 ± 2.4	129.0 ± 2.6
60+ years ‡	140.6 ± 5.0	143.9 ± 2.7	142.2 ± 2.5	146.7 ± 2.9	143.5 ± 3.6	148.7 ± 2.4	140.4 ± 1.8	136.0 ± 1.8
**Non-HDL-C (mg/dL)**								
Age-adjusted ‡	141.1 ± 0.8	140.0 ± 0.6	140.6 ± 0.6	140.0 ± 0.5	137.0 ± 0.7	136.9 ± 0.5	136.0 ± 0.4	133.1 ± 0.4
20–29 years	128.9 ± 2.2	124.3 ± 1.4	125.9 ± 1.6	124.3 ± 1.3	115.7 ± 1.5	115.2 ± 1.1	114.3 ± 1.0	115.2 ± 1.0
30–39 years	141.6 ± 1.6	143.3 ± 1.0	145.8 ± 1.2	146.1 ± 1.3	124.3 ± 1.2	124.0 ± 0.8	125.2 ± 0.9	123.5 ± 0.8
40–49 years	149.7 ± 1.7	149.2 ± 1.2	148.1 ± 1.1	150.2 ± 1.1	135.9 ± 1.2	134.8 ± 0.8	136.6 ± 1.0	133.9 ± 0.9
50–59 years ‡	146.5 ± 1.8	144.5 ± 1.3	146.8 ± 1.3	144.5 ± 1.2	153.6 ± 1.9	153.8 ± 1.2	153.3 ± 1.0	148.4 ± 0.9
60+ years **‡	140.0 ± 1.8	139.0 ± 1.1	136.4 ± 0.8	135.2 ± 0.9	155.7 ± 1.5	157.1 ± 1.1	150.4 ± 0.8	145.4 ± 0.9

*p* for trend were determined by linear regression analysis with weighting of survey design. Abbreviations: KNHANES: Korean National Health and Nutrition Examination Survey; TC: total cholesterol; HDL-C: high-density lipoprotein-cholesterol; TG: triglyceride. Ⅲ: 2005, Ⅳ: 2007–2009, Ⅴ: 2010–2012, Ⅵ: 2013–2015. Bonferroni correction for multiple testing was conducted; ** *p* for trend < 0.00417 in men, ‡ *p* for trend < 0.00417 in women.

**Table 4:** Trends of age-adjusted estimated lipid levels with or without use of lipid-lowering medication according to sex.

KNHANES Phase	III	IV	V	VI	P value	β-coefficient	*P* for Trend
**Men**							
**With use of lipid-lowering medication**							
TC (mg/dL)	195.7 ± 3.0	185.7 ± 0.1	183.2 ± 0.1	174.6 ± 0.1	<0.001	−6.260	<0.001
TG (mg/dL)	240.4 ± 18.5	209.5 ± 0.6	220.6 ± 0.8	213.0 ± 1.0	<0.001	−2.173	0.558
HDL-C (mg/dL)	38.2 ± 0.6	43.6 ± 0.0	44.0 ± 0.0	45.4 ± 0.0	<0.001	1.431	<0.001
Non-HDL-C (mg/dL)	157.5 ± 3.1	142.1 ± 0.1	139.2 ± 0.1	129.1 ± 0.1	<0.001	−7.691	<0.001
**Without use of lipid-lowering medication**							
TC (mg/dL)	183.6 ± 0.9	185.8 ± 0.6	188.2 ± 0.6	188.9 ± 0.5	<0.001	1.735	<0.001
TG (mg/dL)	155.0 ± 3.6	152.3 ± 1.6	153.2 ± 2.1	162.8 ± 2.1	0.004	3.446	0.004
HDL-C (mg/dL)	42.4 ± 0.3	45.5 ± 0.2	46.9 ± 0.2	47.6 ± 0.2	<0.001	1.451	<0.001
Non-HDL-C (mg/dL)	141.2 ± 0.8	140.3 ± 0.6	141.3 ± 0.6	141.3 ± 0.5	ns	0.282	ns
**Women**							
**With use of lipid-lowering medication**							
TC (mg/dL)	195.2 ± 2.4	184.9 ± 0.0	175.4 ± 0.0	172.0 ± 0.0	<0.001	−6.331	<0.001
TG (mg/dL)	172.6 ± 7.7	153.2 ± 0.0	139.3 ± 0.1	136.0 ± 0.1	<0.001	−8.775	<0.001
HDL-C (mg/dL)	46.1 ± 1.2	50.9 ± 0.0	51.8 ± 0.0	54.7 ± 0.0	<0.001	2.224	<0.001
Non-HDL-C (mg/dL)	149.1 ± 1.8	133.9 ± 0.0	123.6 ± 0.0	117.4 ± 0.0	<0.001	−8.555	<0.001
**Without use of lipid-lowering medication**							
TC (mg/dL)	184.4 ± 0.7	188.1 ± 0.5	190.0 ± 0.5	190.1 0.5	<0.001	1.593	<0.001
TG (mg/dL)	112.9 ± 1.5	113.1 ± 1.1	111.4 ± 1.1	109.2 1.0	ns	−1.520	0.010
HDL-C (mg/dL)	47.3 ± 0.2	50.7 ± 0.2	52.7 ± 0.2	54.8 0.2	<0.001	2.300	<0.001
Non-HDL-C (mg/dL)	137.1 ± 0.7	137.4 ± 0.5	137.3 ± 0.4	135.3 0.4	0.012	−0.708	0.006

*P* for trend were determined by age-adjusted linear regression analysis with weighting of survey design. Abbreviations: KNHANES: Korean National Health and Nutrition Examination Survey; TC: total cholesterol; HDL-C: high-density lipoprotein-cholesterol; TG: triglyceride; NS: no significance. Ⅲ: 2005, Ⅳ: 2007–2009, Ⅴ: 2010–2012, Ⅵ: 2013–2015. Bonferroni correction for multiple testing was conducted; significant p-value < 0.0125 and p-for trend < 0.0125.

## References

[1] Benjamin E.J., Blaha M.J., Chiuve S.E., Cushman M., Das S.R., Deo R., de Ferranti S.D., Floyd J., Fornage M., Gillespie C. (2017). Heart disease and stroke statistics-2017 update: A report from the American heart association. Circulation.

[2] World Health Organization Cardiovascular Disease (cvds). http://www.who.int/mediacentre/factsheets/fs317/en/.

[3] World Health Organization (2013). Global Action Plan for the Prevention and Control of Noncommunicable Diseases 2013–2020.

[4] Zabetakis I., Lordan R., Tsoupras A. (2019). The Impact of Nutrition and Statins on Cardiovascular Diseases.

[5] Hong K.N., Fuster V., Rosenson R.S., Rosendorff C., Bhatt D.L. (2017). How low to go with glucose, cholesterol, and blood pressure in primary prevention of cvd. J. Am. Coll. Cardiol..

[6] Navar-Boggan A.M., Peterson E.D., D’Agostino R.B., Neely B., Sniderman A.D., Pencina M.J. (2015). Hyperlipidemia in early adulthood increases long-term risk of coronary heart disease. Circulation.

[7] US Department of Health and Human Services Healthy People 2020 Topics and Objectives: Heart Disease and Stroke. https://www.healthypeople.gov/2020/topics-objectives/topic/heart-disease-and-stroke/objectives..

[8] Carroll M.D., Fryar C.D., Kit B.K. (2015). Total and High-Density Lipoprotein Cholesterol in Adults: United States, 2011–2014.

[9] Wilkins E.W.L., Wickramasinghe K., Bhatnagar P., Leal J., Luengo-Fernandez R., Burns R., Rayner M., Townsend N. (2017). European Cardiovascular Disease Statistics 2017.

[10] Hata J., Ninomiya T., Hirakawa Y., Nagata M., Mukai N., Gotoh S., Fukuhara M., Ikeda F., Shikata K., Yoshida D. (2013). Secular trends in cardiovascular disease and its risk factors in Japanese: Half-century data from the hisayama study (1961–2009). Circulation.

[11] Bayram F., Kocer D., Gundogan K., Kaya A., Demir O., Coskun R., Sabuncu T., Karaman A., Cesur M., Rizzo M. (2014). Prevalence of dyslipidemia and associated risk factors in Turkish adults. J. Clin. Lipidol..

[12] Yang W., Xiao J., Yang Z., Ji L., Jia W., Weng J., Lu J., Shan Z., Liu J., Tian H. (2012). Serum lipids and lipoproteins in Chinese men and women. Circulation.

[13] Nam G.E., Han K., Park Y.G., Choi Y.S., Kim S.M., Ju S.Y., Ko B.J., Kim Y.H., Kim E.H., Cho K.H. (2015). Trends in lipid profiles among south Korean adults: 2005, 2008 and 2010 Korea national health and nutrition examination survey. J. Public Health.

[14] Kweon S., Kim Y., Jang M.J., Kim Y., Kim K., Choi S., Chun C., Khang Y.H., Oh K. (2014). Data resource profile: The Korea national health and nutrition examination survey (knhanes). Int. J. Epidemiol..

[15] Kim Y. (2014). The Korea national health and nutrition examination survey (knhanes): Current status and challenges. Epidemiol. Health.

[16] Expert Panel on Detection, Evaluation (2001). Executive summary of the third report of the national cholesterol education program (ncep) expert panel on detection, evaluation, and treatment of high blood cholesterol in adults (adult treatment panel iii). Jama.

[17] Blaha M.J., Blumenthal R.S., Brinton E.A., Jacobson T.A. (2008). The importance of non–hdl cholesterol reporting in lipid management. J. Clin. Lipidol..

[18] Boekholdt S.M., Arsenault B.J., Mora S., Pedersen T.R., LaRosa J.C., Nestel P.J., Simes R.J., Durrington P., Hitman G.A., Welch K.M. (2012). Association of ldl cholesterol, non-hdl cholesterol, and apolipoprotein b levels with risk of cardiovascular events among patients treated with statins: A meta-analysis. Jama.

[19] Shepherd J., Cobbe S.M., Ford I., Isles C.G., Lorimer A.R., MacFarlane P.W., McKillop J.H., Packard C.J. (1995). Prevention of coronary heart disease with pravastatin in men with hypercholesterolemia. West of Scotland coronary prevention study group. New Engl. J. Med..

[20] Ahn E., Shin D.W., Yang H.K., Yun J.M., Chun S.H., Suh B., Lee H., Son K.Y., Cho B. (2015). Treatment gap in the national health-screening program in Korea: Claim-based follow-up of statin use for sustained hypercholesterolemia. J. Korean Med. Sci..

[21] Dehmer S.P., Maciosek M.V., LaFrance A.B., Flottemesch T.J. (2017). Health benefits and cost-effectiveness of asymptomatic screening for hypertension and high cholesterol and aspirin counseling for primary prevention. Ann. Fam. Med..

[22] Grundy S., Becker D., Clark L., Cooper R., Denke M., Howard J., Hunninghake D., Illingworth D., Luepker R., McBride P. (2002). Detection, evaluation, and treatment of high blood cholesterol in adults (adult treatment panel iii). Circulation.

[23] Helfand M., Carson S.U.S. (2008). Preventive services task force evidence syntheses, formerly systematic evidence reviews. Screening for Lipid Disorders in Adults: Selective Update of 2001 us Preventive Services Task Force Review.

[24] Smith S.C., Grundy S.M. (2014). 2013 acc/aha guideline recommends fixed-dose strategies instead of targeted goals to lower blood cholesterol. J. Am. Coll. Cardiol..

[25] Yokoyama S. (2018). Trend of hdl increase among Japanese people continues in national health and nutrition survey. J. Atheroscler. Thromb..

[26] Pedram P., Aref-Eshghi E., Mariathas H.H., Hurley O., Godwin M., Duke P., Mahdavian M., Asghari S. (2018). Six-year time-trend analysis of dyslipidemia among adults in newfoundland and labrador: Findings from the laboratory information system between 2009 and 2014. Lipids Health Dis..

[27] Kim S.M., Han J.H., Park H.S. (2006). Prevalence of low hdl-cholesterol levels and associated factors among koreans. Circ. J. Off. J. Jpn. Circ. Soc..

[28] Roth G.A., Johnson C., Abajobir A., Abd-Allah F., Abera S.F., Abyu G., Ahmed M., Aksut B., Alam T., Alam K. (2017). Global, regional, and national burden of cardiovascular diseases for 10 causes, 1990 to 2015. J. Am. Coll. Cardiol..

[29] Shin H.Y., Kang H.T. (2017). Recent trends in the prevalence of underweight, overweight, and obesity in Korean adults: The Korean national health and nutrition examination survey from 1998 to 2014. J. Epidemiol..

[30] Verschuren W.M., Jacobs D.R., Bloemberg B.P., Kromhout D., Menotti A., Aravanis C., Blackburn H., Buzina R., Dontas A.S., Fidanza F. (1995). Serum total cholesterol and long-term coronary heart disease mortality in different cultures. Twenty-five-year follow-up of the seven countries study. Jama.

[31] Luepker R.V., Johnson S.B., Breslow L., Chobanian A.V., Davis C.E., Duling B.R., Oparil S. (1996). Physical activity and cardiovascular health. Nih consensus development panel on physical activity and cardiovascular health. Jama.

[32] Juonala M., Magnussen C.G., Berenson G.S., Venn A., Burns T.L., Sabin M.A., Srinivasan S.R., Daniels S.R., Davis P.H., Chen W. (2011). Childhood adiposity, adult adiposity, and cardiovascular risk factors. New Engl. J. Med..

[33] Cho I.Y., Park H.Y., Lee K., Bae W.K., Jung S.Y., Ju H.J., Song J.K., Han J.S. (2017). Association between the awareness of dyslipidemia and health behavior for control of lipid levels among Korean adults with dyslipidemia. Korean J. Fam. Med..

[34] Scandinavian Simvastatin Survival Study Group (1994). Randomised trial of cholesterol lowering in 4444 patients with coronary heart disease: The scandinavian simvastatin survival study (4s). Lancet.

[35] Pekkanen J., Linn S., Heiss G., Suchindran C.M., Leon A., Rifkind B.M., Tyroler H.A. (1990). Ten-year mortality from cardiovascular disease in relation to cholesterol level among men with and without preexisting cardiovascular disease. New Engl. J. Med..

[36] Tsoupras A., Lordan R., Zabetakis I. (2018). Inflammation, not cholesterol, is a cause of chronic disease. Nutrients.

[37] Martin S.S., Blaha M.J., Elshazly M.B., Brinton E.A., Toth P.P., McEvoy J.W., Joshi P.H., Kulkarni K.R., Mize P.D., Kwiterovich P.O. (2013). Friedewald-estimated versus directly measured low-density lipoprotein cholesterol and treatment implications. J. Am. Coll. Cardiol..

[38] Moriyama K., Takahashi E. (2016). Non-hdl cholesterol is a more superior predictor of small-dense ldl cholesterol than ldl cholesterol in Japanese subjects with tg levels 400 mg/dl. J. Atheroscler. Thromb..

[39] Arsenault B.J., Rana J.S., Stroes E.S., Despres J.P., Shah P.K., Kastelein J.J., Wareham N.J., Boekholdt S.M., Khaw K.T. (2009). Beyond low-density lipoprotein cholesterol: Respective contributions of non-high-density lipoprotein cholesterol levels, triglycerides, and the total cholesterol/high-density lipoprotein cholesterol ratio to coronary heart disease risk in apparently healthy men and women. J. Am. Coll. Cardiol..

[40] Drexel H. (2009). Statins, fibrates, nicotinic acid, cholesterol absorption inhibitors, anion-exchange resins, omega-3 fatty acids: Which drugs for which patients?. Fundam. Clin. Pharmacol..

[41] Doonan R., Hafiane A., Lai C., Veinot J., Genest J., Daskalopoulou S. (2014). Cholesterol efflux capacity, carotid atherosclerosis, and cerebrovascular symptomatology. Arterioscler. Thromb. Vasc. Biol..

[42] Khera A.V., Demler O.V., Adelman S.J., Collins H.L., Glynn R.J., Ridker P.M., Rader D.J., Mora S. (2017). Cholesterol efflux capacity, high-density lipoprotein particle number, and incident cardiovascular events: An analysis from the Jupiter trial (justification for the use of statins in prevention: An intervention trial evaluating rosuvastatin). Circulation.

